# Reorganising specialist cancer surgery for the twenty-first century: a mixed methods evaluation (RESPECT-21)

**DOI:** 10.1186/s13012-016-0520-5

**Published:** 2016-11-25

**Authors:** Naomi J. Fulop, Angus I. G. Ramsay, Cecilia Vindrola-Padros, Michael Aitchison, Ruth J. Boaden, Veronica Brinton, Caroline S. Clarke, John Hines, Rachael M. Hunter, Claire Levermore, Satish B. Maddineni, Mariya Melnychuk, Caroline M. Moore, Muntzer M. Mughal, Catherine Perry, Kathy Pritchard-Jones, David C. Shackley, Jonathan Vickers, Stephen Morris

**Affiliations:** 1 Department of Applied Health Research, University College London, 1-19 Torrington Place, London, WC1E 7HB UK; 2 Royal Free London NHS Foundation Trust, London, UK; 3 Alliance Manchester Business School, University of Manchester, Manchester, UK; 4 Patient representative, London, UK; 5 Research Department of Primary Care and Population Health, University College London, London, UK; 6 University College London Hospitals NHS Foundation Trust, London, UK; 7 Barts Health NHS Trust, London, UK; 8 Salford Royal NHS Foundation Trust, Salford, UK; 9 Division of Surgery and Interventional Science, University College London, London, UK

**Keywords:** Cancer, Improvement science, Service reorganisation, Organisational change, Implementation, Health services research, Discrete choice experiment, Cost-effectiveness

## Abstract

**Background:**

There are longstanding recommendations to centralise specialist healthcare services, citing the potential to reduce variations in care and improve patient outcomes. Current activity to centralise specialist cancer surgical services in two areas of England provides an opportunity to study the planning, implementation and outcomes of such changes. *London Cancer* and *Manchester Cancer* are centralising specialist surgical pathways for prostate, bladder, renal, and oesophago-gastric cancers, so that these services are provided in fewer hospitals. The centralisations in London were implemented between November 2015 and April 2016, while implementation in Manchester is anticipated in 2017.

**Methods/Design:**

This mixed methods evaluation will analyse stakeholder preferences for centralisations; it will use qualitative methods to analyse planning, implementation and sustainability of the centralisations (‘how and why?’); and it will use a controlled before and after design to study the impact of centralisation on clinical processes, clinical outcomes, cost-effectiveness and patient experience (‘what works and at what cost?’). The study will use a framework developed in previous research on major system change in acute stroke services. A discrete choice experiment will examine patient, public and professional preferences for centralisations of this kind. Qualitative methods will include documentary analysis, stakeholder interviews and non-participant observations of meetings. Quantitative methods will include analysis of local and national data on clinical processes, outcomes, costs and National Cancer Patient Experience Survey data. Finally, we will hold a workshop for those involved in centralisations of specialist services in other settings to discuss how these lessons might apply more widely.

**Discussion:**

This multi-site study will address gaps in the evidence on stakeholder preferences for centralisations of specialist cancer surgery and the processes, impact and cost-effectiveness of changes of this kind. With increasing drives to centralise specialist services, lessons from this study will be of value to those who commission, organise and manage cancer services, as well as services for other conditions and in other settings. The study will face challenges in terms of recruitment, the retrospective analysis of some of the changes, the distinction between primary and secondary outcome measures, and obtaining information on the resources spent on the reconfiguration.

## Background

### Centralising services to improve quality of care and patient outcomes

There are longstanding recommendations in the English National Health Service (NHS) and internationally to centralise specialist services [1–5]. Recent guidance in the English NHS indicates that centralising specialist services will remain a priority in the future [6, 7]. Centralisation has potential to improve care provision and patient outcomes by increasing the likelihood of patients being treated in hospitals that have a full range of experienced specialists and equipment to support care provision. For instance, recent research indicates that centralising acute stroke services into fewer high-volume units is associated with significantly better provision of evidence-based clinical interventions [8] and significantly greater reductions in patient mortality [9]. However, little is known about processes by which services are centralised, the impact of changes on patients and staff, and factors influencing implementation [10].

### Centralising cancer services

Recent research indicates that there is limited evidence of the cost impact of centralising cancer services [10, 11] and patient, public and professional preferences in relation to centralisations of this kind [12, 13]. High volume is associated with better outcomes in specialist surgery for OG cancers [14] and urological cancers [15]. However, the strength of this relationship varies between specialties [16]. Furthermore, centralising cancer services may place increased travel demands on patients and families and may limit people’s access to quality care [17]. A review of evidence indicates that willingness to travel for specialist care is greater if a hospital has a good reputation, if the condition is serious or urgent and if the patient is of a higher socioeconomic status; willingness to travel further is lower amongst older patients and frequent users of services, and preferences vary according to the length of the journey [18, 19].

### Specialist surgical services for urological and oesophago-gastric cancers in London Cancer and Manchester Cancer

Networked cancer systems London Cancer (covering the geographical areas of North Central London, North East London, and West Essex (population 3.2 million)) and Manchester Cancer (covering Greater Manchester and East Cheshire (population 3.1 million)) have been working towards centralising specialist surgery services for a number of cancers [20, 21]. This study will evaluate four of the surgical cancer pathways that are being centralised in both areas: prostate, renal, bladder and oesophago-gastric (OG) cancers. There are over 60,000 new cases of these cancers in the UK every year [22–25]. Prostate cancer is the second highest cause of cancer deaths in men [23]. Five-year survival rates are 85% for prostate cancer [26], 50–60% for bladder and renal cancers [22, 24], 12% for oesophageal cancer and 16% for gastric cancer [3].

### Pre-centralisation pathways

**Fig. 1:**
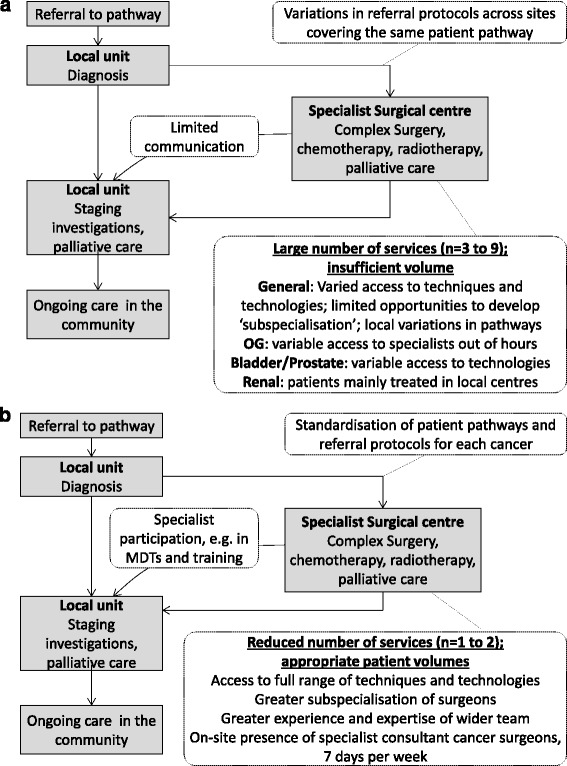
Simplified models summarising specialist cancer surgery—(*1a*) before and (*1b*) after centralisation

In both areas, pre-centralisation, potential cancer patients were referred to their local cancer centre for diagnosis, and either remained there for treatment or were referred to a specialist centre; protocols for referral to specialist centres varied across referring sites. The range of treatment available to patients (e.g. access to robotic surgery for prostate and bladder patients; access to specialist surgeon for renal and OG patients; degree of subspecialisation of urological surgeons) varied significantly between specialist centres, as did patient volumes (Fig. 1a).

### Centralisations in London Cancer and Manchester Cancer

London Cancer and Manchester Cancer have proposed that specialist surgical services for each of these cancers should be centralised into fewer specialist centres (Table 1), with standardised patient pathways, with the aim of reducing variations in care. It is anticipated that increased patient volumes in specialist centres will allow greater specialisation of staff and greater experience and expertise across teams working in those centres [27, 28]. Further, specialist centres will offer a full range of surgical technologies (e.g. robotics) and equal access to innovative techniques, including less invasive surgical procedures and non-surgical procedures (such as radiotherapy, brachytherapy and hormone therapy) [27, 29]. These centralisations are being conducted within a wider context of change, impacting on specialist services for other cancers and other health conditions, e.g. cardiac services. In some cases, the changes build on previous activity to centralise specialist cancer surgery into a smaller number of services.

**Table 1:** Overview of services providing specialist surgery: mean number of cases per year, number requiring complex surgery per year and number of specialist centres pre- and post-centralisation (2015)

Cancer	London Cancer				Manchester Cancer			
	Total cases	Require surgery	Specialist centres		Total cases	Require surgery	Specialist centres	
			Before	After			Before	After
Bladder	372	130	2	1	628	113	5	↓
Prostate	1600	220	2	1	1879	283	5	↓
Renal	282	190	9	1	407	269	5	↓
OG	566	129	3	2	868	152	3	↓

London Cancer figures [20, 25]; Manchester Cancer figures [21, 25]; number of post-centralisation centres in Manchester Cancer still to be confirmed

Post-centralisation, local units will continue to provide much patient care, including diagnosis, ongoing radiotherapy, chemotherapy and some forms of non-complex surgery. In addition, it is planned that they will have closer involvement with specialist centres, with the aim of improving quality of care across the whole system, through participation in specialist multidisciplinary teams (SMDTs), and specialists providing training and delivering some outpatient care. Both sets of proposals emphasise the importance of continuity of care for patients (in relation to coordination of care and ongoing contact with patients across different services over time), for example by the specialist centre hosting follow-up clinics and joint appointments in local units [20, 21].

### Current status of centralisations

Implementation of the London Cancer centralisations was completed over the period November 2015 to April 2016. Implementation of the Manchester Cancer centralisations is planned for 2017.

### Conceptual framework

**Fig. 2:**
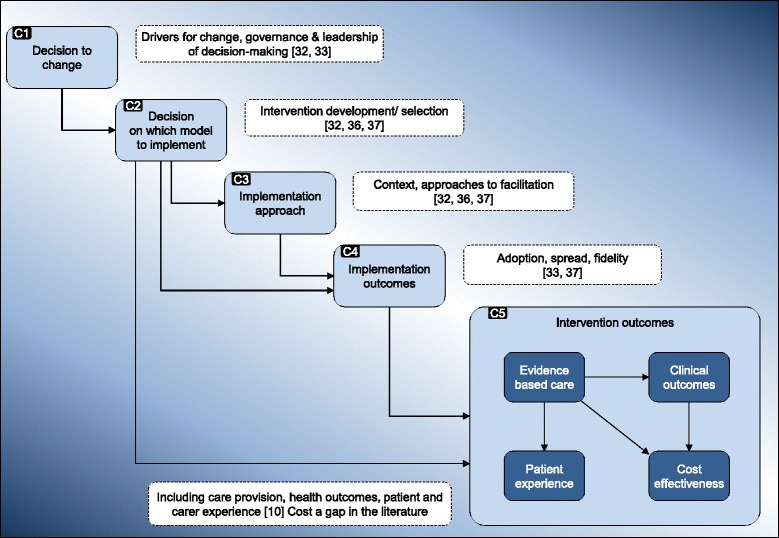
Conceptual framework: key components of major system change [31]

The study will use a framework developed as part of a study of major system change in acute stroke services and designed to be applicable in various clinical settings [30, 31] (Fig. 2). In this study, we will further develop this framework in a different healthcare context; in addition, as the centralisations have been led by networked systems, we will consider how networks influence major system change. Our framework presents key processes of major system change and the relationships between them: the decision to change (C1) (drivers for change, and how the decision to change is led and governed, e.g. the importance of combining ‘bottom up’ clinical leadership with ‘top down’ central leadership, the impact of social and political context [32, 33] and the role played by clinical leaders in sharing knowledge and driving change across networks [34, 35]); developing and agreeing new service models (C2) (e.g. clinicians working across organisational boundaries to develop service specifications) [32, 36, 37]; how changes are implemented (C3) (e.g. the degree to which implementation is phased, the level of hands-on facilitation and ways in which knowledge is shared across organisational and professional boundaries) [32, 36–38]; adherence to the new model (C4) (including factors that might influence fidelity to the new pathways, e.g. pathway complexity and organisational boundaries) [33, 37, 38].

This evaluation was peer reviewed by the National Institute for Health Research Health Services and Delivery Research (NIHR HS&DR) Programme prior to being funded (project ref 14/46/19) and received ethical approval in July 2015 from the Proportionate Review Sub-committee of the NRES Committee Yorkshire and the Humber—Leeds (reference 15/YH/0359).

### Study aim, research questions

This study aims to analyse centralisation of specialist cancer surgery services in two areas of England (covered by London Cancer and Manchester Cancer) and identify lessons to inform centralisation in other healthcare settings. It will address the following research questions:

1. What are patient, public and professional preferences in relation to these centralisations?
2. What were the key processes in centralising specialist cancer surgery services in the two regions?
3. What is the impact of the centralisations on staff and healthcare provider organisations, including ways of working, skill mix and approaches to collaboration?
4. What is the impact on provision of care, in terms of clinical processes and outcomes?
5. What is the impact on patient experience, including choice and continuity of care?
6. What is the cost and cost-effectiveness of the changes?
7. How might lessons from centralising specialist cancer surgery services be applied in future centralisations of specialist cancer services and other specialist settings?

## Methods/Design

### Design

This is a multi-site, contemporaneous study of the centralisation of specialist surgical pathways for four cancers in two large conurbations in England. To understand stakeholder preferences for the organisation of cancer specialist surgical services, a discrete choice experiment will be conducted; to analyse the centralisations, we will combine quantitative analysis of the impact of centralisation on clinical processes, clinical outcomes, cost-effectiveness and patient experience (‘what works and at what cost?’), with qualitative analysis of their development, implementation and sustainability (‘how and why?’); a similar approach was used previously in a study of major system change in acute stroke services [30].

The study will focus on four surgical pathways (for prostate, bladder, renal and OG cancers). These pathways have been selected because they are being centralised in both areas, facilitating analysis of how changes occur in different contexts. The study will also analyse different degrees of centralisation, as the reduction in number of specialist centres varies significantly (see Table 1).

#### Understanding stakeholder preferences

We will conduct a discrete choice experiment (DCE) [39–41] to examine stakeholder (patients, the public, healthcare professionals) preferences for centralisation, in relation to such attributes as travel time to hospital to undergo surgery, and risk of serious complications.

#### Understanding implementation and sustainability

We will use documentary analysis, stakeholder interviews and non-participant observations to identify drivers for change; how the centralisations were planned and implemented and factors influencing this; the extent to which the proposals were implemented; and factors influencing sustainability of the changes.

#### Understanding what works and at what cost

This component of the evaluation will use a controlled before and after design. The centralisations will be analysed in terms of the extent to which changes were implemented and the impact of centralisation on care provision, clinical outcomes, patient experience and cost-effectiveness.

#### Exploring generalisability to other contexts

To develop lessons that might support centralisation in other contexts, we will host a workshop for stakeholders involved in planning centralisations of specialist cancer services elsewhere and other types of specialist service. Stakeholders will include providers, commissioners and patients and patient groups from across the country. Working with attendees, we will identify factors influencing generalisability of our findings and develop lessons that will be of use in different settings.

### Sampling

#### Discrete choice experiment

The DCE will elicit preferences for how services are organised from three stakeholder groups: patients, the public and healthcare professionals. Each stakeholder group will sample London, Greater Manchester and elsewhere in England. Sample size calculations for DCEs are not straightforward but a sample size of 300 is commonly recommended [42]. Our sample size will be 400:200 patients with prostate, bladder, renal and OG cancers, (25% for each cancer); 100 healthcare professionals involved in managing these cancers; and 100 members of the public (anyone who is neither a cancer patient nor a healthcare professional).

#### Documentary analysis, stakeholder interviews and non-participant observations

We will collect documents covering development, planning and implementation of the centralisations. We will also collect documents on contextual factors, including policy and media coverage. Documents will be collected from 2006, when the Royal College of Surgeons of England launched a consultation on centralisation of surgical services [43].

**Fig. 3:**
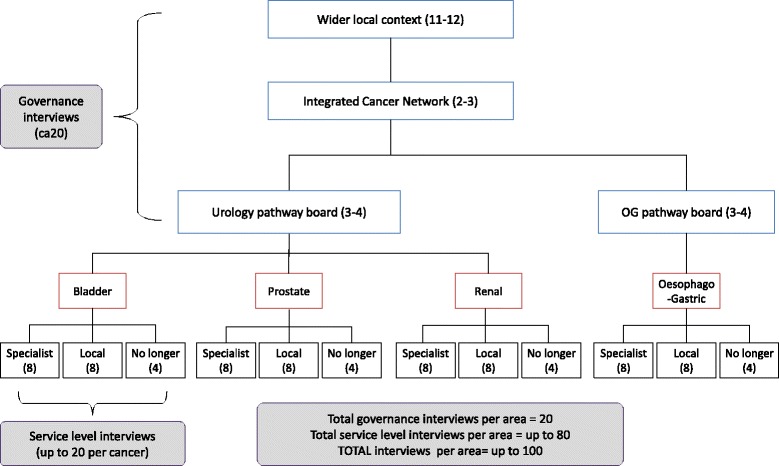
Anticipated interviewee recruitment per area

We will sample up to 200 stakeholder interviewees purposively across London Cancer and Manchester Cancer (Fig. 3). Participants will include people involved with governance of the centralisations (e.g. programme boards, local commissioners and patient representative groups). For each cancer pathway, we will interview clinicians and managers in a specialist centre, a local unit and a service no longer providing specialist surgical care.

Researchers will observe activities related to planning, implementation and ongoing governance of the centralised services, for example pathway board meetings, multidisciplinary team meetings and training events.

#### Impact on clinical processes, outcomes and patient experience

Table 2 summarises the data we will analyse and Table 3 the key variables.

**Table 2:** Summary of datasets

Dataset	Year change occurs	Years sampled	Mean number of patients per year, by area	Notes
Prostate cancer				
National Prostate Cancer Audit	2015	2014–2017	*Incidence of prostate cancer:* London Cancer = 1600 Manchester Cancer = 1879 Rest of England = 30,637	Audit commenced 2014 Mean annual incidence of prostate cancer from UK Cancer Atlas data 2008–2010 [25]
True NTH UK—post surgical follow-up	2015	2014–2017	London Cancer = 500 Manchester Cancer = 300 Rest of England = 500	Estimated figures from co-author CMM (True NTH UK—post surgical follow-up project lead)
National Cancer Patient Experience Survey	2015	2014–2017	London Cancer = 276 Manchester Cancer = 307 Rest of England = 5002	National Cancer Patient Experience Survey (2013) [65]
BAUS Radical prostatectomy dataset	2015	2014–2017	BAUS audit participation (national) = 2093	BAUS Radical Prostatectomy Audit report (2012) [66]
Bladder cancer				
Hospital episode statistics	2015	2014–2017	Patients undergoing cystectomy (national) = 1360 *Incidence of bladder cancer:* London Cancer = 628 Manchester Cancer = 372 Rest of England = 7895	From NCIN analysis of 2005–2007 bladder cystectomies Mean annual incidence of bladder cancer from UK Cancer Atlas data 2008–2010 [25, 67]
National Cancer Patient Experience Survey	2015	2014–2017	London Cancer = 321 Manchester Cancer = 410 Rest of England = 6327	National Cancer Patient Experience survey (2013) [65] NB these are overall figures for urological cancers—will be disaggregated by ICD10 code.
Renal cancer				
BAUS audit of nephrectomies	2015	2014–2017	BAUS audit participation = 5851 *Incidence of renal cancer:* London Cancer = 282 Manchester Cancer = 407 Rest of England = 5930	From BAUS nephrectomy audit report (2012) Mean annual incidence of renal cancer from UK Cancer Atlas data 2008–2010 [25]
National Cancer Patient Experience Survey	2015	2014–2017	London Cancer = 321 Manchester Cancer = 410 Rest of England = 6327	National Cancer Patient Experience survey (2013) [65] NB these are overall figures for urological cancers – will be disaggregated by ICD10 code.
OG cancer				
AUGIS national audit	2015	2014–2017	Patients undergoing oesophagectomy and gastrectomy (England) = 1967 *Incidence of OG cancer*: London Cancer = 868 Manchester Cancer = 566 Rest of England = 11529	From AUGIS OG audit report (2013) Mean annual incidence of OG cancer from UK Cancer Atlas data 2008–2010 [25, 68]
National Cancer Patient Experience Survey	2015	2014–2017	London Cancer = 221 Manchester Cancer = 202 Rest of England = 3860	National Cancer Patient Experience survey (2013) [65] NB these are overall figures for upper GI cancers – will be disaggregated by ICD10 code.

**Table 3:** Summary of primary and secondary outcomes, process measures, mediating factors and required sample sizes for each cancer

Prostate cancer	
Primary outcome	• Radical prostatectomy: proportion of men treated by primary surgery who remain continent (pad free) at 12 months (research indicates range of 80–92%, depending on procedure) [69]
Secondary outcomes	• Radical prostatectomy: proportion of men treated by surgery with pre-operative erectile function who have erections sufficient for penetration at 12 months • Length of stay • Readmission • Surgical complications • Post-operative complications • Diagnostic outcomes: proportion of men diagnosed with clinically significant prostate cancer • Patient experience, including choice of treatment, access to services, confidence in staff, communication, effectiveness of teamwork and opportunity to participate in research
Bladder cancer	
Primary outcome	• 30-day post-operative mortality (national figure (2012) = 2.4%) [70]
Secondary outcomes	• Length of stay • Proportion of patients offered neo-bladder reconstruction • Proportion of patients receiving neo-bladder reconstruction • Surgical complications (measured by Clavien–Dindo grading) • Patient experience (measures as above)
Renal cancer	
Primary outcome	• 30-day post-operative mortality (anticipated figure = 0.9%) [71]
Secondary outcomes	• 30 day readmission • % of cases of T1a tumours having nephron-sparing surgery • Length of stay • Surgical complications (measured by Clavien–Dindo grading) • Conversion from laparoscopic (including robotically assisted) to open surgery • Patient experience (measures as above)
OG cancer	
Primary outcome	• 30-day post-operative mortality (national figure (2013) = 2.3%) [72]
Secondary outcomes	• % of patients offered endoscopic resection for tumours staged as T1a • Length of stay • % Complete R0 resection (i.e. full removal of tumour) • Surgical complications—anastomotic leak • Patient experience (measures as above)
Intermediate outcomes (all)	
	• Waiting times (within 62 days of referral, 31 days of decision to treat) • Number of patients seen by surgeon • Case volume per surgeon • Proportion of cases where surgery is an emergency procedure
Mediating factors (all)	
	• Patient characteristics (age, gender, ethnicity, socioeconomic status) • Cancer stage • Whether procedure is a salvage procedure

The outcomes denoted as primary in Table 3 were selected with clinical collaborators to enable sample size calculations to demonstrate the feasibility of the quantitative analysis. However, we note that selecting a single measure as being of primary importance in a mixed methods evaluation of a complex intervention is difficult, as a range of outcomes is likely to be important to stakeholders. Consequently, our study will analyse a range of outcome measures and consider the distinction between ‘primary’ and ‘secondary’ measures to be somewhat arbitrary.

For prostate cancer, we need a sample size of 1074 patients comprising 119 in the exposed group (London Cancer and Manchester Cancer post-reconfiguration) and 955 in the unexposed group (rest of England plus London Cancer and Manchester Cancer pre-reconfiguration) to have an 80% chance of detecting, as significant at the 5% level, an increase in the proportion of men treated by primary surgery who are continent at 12 months from 80 to 90% assuming an enrolment ratio of 8:1 (http://clincalc.com/stats/samplesize.aspx [26 September 2016]). For bladder, renal and OG cancers, we need a sample size of 4446 patients (494 in the exposed group) for the same statistical power and enrolment ratio as above to detect a decrease in 30-day post-operative mortality from 2 to 1%. Based on the figures in Table 2, we expect our datasets to contain information on primary outcomes for approximately the following number of patients per year: prostate cancer, 1300; bladder cancer, 1400; renal cancer, 6000; OG cancer, 2000. For each cancer, we will have at least 4 years of data (2014–2017), suggesting that the required sample sizes are feasible.

#### Cost-effectiveness

Collaborating with providers and commissioners, we will obtain information on the costs associated with planning and implementing the centralisations. Some of these costs are likely to represent one-off, sunk costs to providers and commissioners, and will be important in informing other organisations about potential cost of centralisation. We will obtain data regarding outcomes of surgery pre- and post-centralisation to allow us to quantify any impact on cost per procedure and in-patient hospital costs that may occur as a result of centralisation, for example from changes in patient case-mix and complexity, changes in bed management practices or how theatres are booked and used. The cost-effectiveness of the centralisations will be reported as incremental cost per quality-adjusted life-year gained and incremental cost per change in outcome. The outcome(s) included in the latter will be those listed in Table 2 plus others informed by the DCE ranking results, for example if stakeholders indicate a clear preference for more than one outcome, a separate analysis will be performed for each.

### Data collection

#### Discrete choice experiment

We will develop a questionnaire that elicits stakeholder preferences for the reorganisation of cancer surgical services. The questionnaire will present choices between options differing on a number of attributes that may be affected by centralisation, for example travel time to hospital for surgery, number of operations the centre conducts annually and the risk of serious complications from surgery. We will identify attributes by reviewing the literature and consulting with patients, the public and professionals. Attribute descriptions will undergo review by the Plain English Campaign. Once identified, we will determine plausible levels of each attribute based on clinically feasible ranges derived from systematic literature reviews, planning documents, audit reports, NICE guidelines, published studies and consultations with healthcare professionals.

To design the questionnaire, we will use a pairwise choice framework and compile a set of pairwise scenarios that describe feasible combinations of levels and attributes of centralised versus non-centralised cancer surgery services. The number of pairwise choices will be reduced to a practical number for participants to answer (eight questions per participant) using an algorithm that maximises the efficiency of the experimental design [44]. The questionnaire will undergo plain English review and will be piloted with patient representatives.

Quality Health (an organisation specialising in conducting surveys in healthcare settings, including the National Cancer Patient Experience Survey (NCPES)) will assist in the distribution and will be in charge of data collection, data entry and preparation of the dataset. All data collected will be anonymised, and demographic details will be categorised so that participants cannot be identified.

#### Documents, stakeholder interviews and non-participant observations

Documentation covering planning, implementation and impact of the centralisations will be obtained from staff in Manchester Cancer, London Cancer and participating provider and commissioning organisations. We will also conduct online searches for further local and national documentation, including relevant policy, guidance and media reports.

Interview topic guides will be developed in collaboration with patient and clinical members of the research team to focus on key aspects of the centralisations, including the decision to change, planning and implementing the changes, perceived impact and sustainability of changes and influential factors (e.g. local and national contexts). Interviews will be digitally recorded for professional transcription.

Non-participant observations will focus on recording decision-making processes, the stakeholders who are involved and not involved in planning and implementation, and influential contextual factors. Observations will be recorded as field notes. All interview and observation data will be stored securely and anonymised.

#### Impact on clinical processes, clinical outcomes and patient experience

We will formally request data from the relevant organisations (Table 2).

#### Cost-effectiveness

To populate the cost-effectiveness models, we will use clinical process, clinical outcomes and patient experience data, along with data from published sources including:

- Probabilities of disease progression (obtained from systematically reviewing epidemiological and other literature);
- Unit costs of healthcare resources used, including professionals’ time (obtained from NHS reference costs [45], previous studies [46], British National Formulary [47], unit costs of Health and Social Care [48]);
- Health state utilities (obtained from the Cost-Effectiveness Analysis Registry) [49].

### Recruitment

#### Discrete choice experiment

The questionnaire will be distributed with a cover letter and participant information sheet: these make clear that consent is implied by participation in the survey.

Quality Health will use the NCPES database to identify cancer patients who have agreed to take part in research. A sample of these patients will be sent the questionnaire and study information by post and invited to return the questionnaire by post or complete it online.

Quality Health will recruit members of the public by advertising the survey through health-related charities’ websites, newsletters, and mailing lists. Advertisements will include a link to the online questionnaire and study information.

To recruit healthcare professionals, the research team will advertise the study through websites, newsletters and mailing lists of relevant groups and organisations in London, Greater Manchester and nationwide. Advertisements will include a link to the online questionnaire and study information.

#### Stakeholder interviews and non-participant observations

Study researchers will approach potential interviewees and share study information via e-mail and telephone. Potential interviewees will have at least 48 h to consider participating and ask any questions about the research. Interviews will be conducted only with written, fully informed consent. Participants will be free to withdraw at any time.

Permission to observe meetings will be obtained from the Chair in advance. Participant information sheets will be circulated with meeting papers to all members. On first attendance, researchers will inform members about the study, what participation entails, and that members may decline to participate at any time. At subsequent meetings, the researcher will announce him/herself as a non-participant observer; agreement for observation to proceed will be recorded in meeting minutes. If participants do not agree to participate, any contributions they make to the meeting will be excluded from field notes; if more appropriate, the researcher will withdraw from the meeting.

Ward observations will only be conducted with permission from clinical staff. Before commencing observations, researchers will attend meetings to discuss the study and obtain staff consent. Patients will not be recruited because researchers will not directly observe patients, but rather staff activity as patients pass through cancer services. Posters explaining the purpose of the observations will be displayed in participating clinical areas, and copies of information sheets will be available. During observations, researchers will be clearly identified with a badge, and will announce their presence.

### Combining methods

Research strands will be combined throughout the lifespan of the study. In relation to data collection, interview topic guides will be informed initially by the documentary analysis and primary measures used in the quantitative analyses; later topic guides will incorporate findings from these analyses. Our document inventory will be structured to facilitate the cost-effectiveness analysis. The process and outcome analyses will in part be guided by documentary analysis (e.g. identifying the ‘before’ and ‘after’ periods of centralisations). Potential sources of cost data will be partly identified through interviews and documentary analysis (e.g. relating to staffing and resource use), while the focus of the cost-effectiveness analysis will be guided by the results of the outcomes analysis and DCE.

### Data analysis

#### Discrete choice experiment

We will summarise respondents’ rankings of attributes descriptively and compare these between the three stakeholder subgroups, testing for differences using rank correlation coefficients. We will analyse preference data using conditional logistic regression analysis. Attributes measured on a continuous scale will be mean-centred; attributes measured as categorical variables will be effects coded. The regression analysis will indicate relative importance of attributes to respondents. Data will be analysed for all respondents jointly and disaggregated by the three stakeholder subgroups, controlling for available baseline demographic characteristics. To explore trade-offs participants make between attributes, we will calculate the marginal rates of substitution (MRS), calculated as a ratio of the coefficients of two attributes. The MRS allows direct assessment of how much of one attribute respondents are willing to trade for one unit of another attribute and enables comparison of different attributes on a common scale [50].

We will use the regression results to calculate the predicted probability that different combinations of attribute levels used in the experiment would be selected. This allows us to rank centralised versus non-centralised services in terms of their order of preference by respondents [50] and to explore how this ranking varies by stakeholder group.

#### Documentary analysis, stakeholder interviews and non-participant observations

The documentary analysis will draw on our conceptual framework (Fig. 2), reflecting key processes of change and influential factors (e.g. governance structure, local and national context). The data from the documentary analysis will contribute to the development of detailed timelines and narrative summaries of the centralisations.

Ongoing and iterative thematic analysis of interview and observation data will be conducted, following established procedures of constant comparative analysis [51]. Initial analysis and category building will include category mapping and constant comparison. A subgroup of co-investigators with qualitative expertise will develop the analysis; and the whole research team will contribute to interpretation of findings. Validity will be assessed in relation to Patton’s four criteria of validity in qualitative research: verification, rival explanations, negative cases and triangulation [52].

#### Impact on clinical processes, clinical outcomes and patient experience

We will aggregate risk-adjusted patient level data by Trust and time (quarter) and use between-region difference-in-differences regression analysis to investigate the impact of the centralisations on clinical processes, clinical outcomes and patient experience.

We will risk-adjust the observed patient outcomes using expected outcomes that are obtained from patient level regression models. For the primary outcomes, which are binary, we will use logistic regression to regress each outcome against a series of covariates including: gender; age; interactions between age and gender; cancer diagnosis; Charlson comorbidity index [53]; presence of 16 comorbidities included in the Charlson comorbidity index; ethnic group; deprivation quintile based on area of residence [54]; and rural-urban classification based on area of residence [55]. The patient level regressions will be run only on patients who had surgery before the reorganisations so the risk adjustment will not be contaminated by the changes. The regression coefficients will be used to predict the probability of the outcome for every patient, in both pre- and post-implementation periods. These will be aggregated to create a dataset of the actual outcomes (actual percentage of patients who were pad free at 12 months or who had died by 30 days) and the expected outcomes, by admitting hospital and quarter (from the logistic regressions).

For each outcome and type of cancer, we will construct a Trust-by-quarter dataset covering the whole of England where possible containing data on clinical outcomes and care processes plus covariates. We will regress the risk-adjusted outcomes, measured at the Trust level in each quarter, against a variable denoting cancer surgery service centralisations, controlling for Trust and time fixed effects. For the aggregate data the regression model is

$$ {y}_{jt}={\alpha}_1+{u}_j+{v}_t+{\delta}_1{D}_{jt}^1{D}_{jt}^2+{e}_{jt} $$

where *y* is the risk-adjusted outcome of interest (mortality, readmissions, LOS; actual minus expected values), *j* indicates Trust, *t* indicates quarter, *α* is a constant term, *u* represents Trust fixed effects and *v* represents time (quarter) fixed effects. *D* ^1^ is a variable taking the value 1 if the provider Trust is in London Cancer/Manchester Cancer and 0 otherwise; *D* ^2^ is a variable which equals 1 if the observation belongs to the time period after the centralisation and 0 otherwise. Sample weights based on patient numbers in each Trust/quarter will be used. We are interested in the sign and statistical significance of the coefficient *δ*_1_, which quantifies the changes in risk-adjusted outcomes over time in London Cancer and Manchester Cancer controlling for the changes over time in the rest of England. We will rerun the analysis using outcomes that are not risk-adjusted at the first stage as a sensitivity analysis. We will run pre-trend tests to examine whether the outcomes had a different linear trend in London Cancer and Manchester Cancer compared with the rest of England before the centralisations.

This two-stage approach (patient level risk adjustment followed by between-region difference-in-differences analysis on aggregate Trust-by-quarter data) is consistent with Medical Research Council guidelines for using natural experiments to evaluate population health interventions [56] and has been used in previous studies [8, 57].

We will repeat the above analysis for the secondary outcomes. The regression model used at the first stage will reflect the functional form of the outcome measure, for example: most of the outcomes are binary measures so we will use logistic regression as described for the primary analysis; length of stay will be analysed using a generalised linear model with gamma family and log link to account for data skewness [58].

We will undertake a secondary analysis using synthetic controls [59–62], defining a control group closely resembling regions in which centralisation occurred in terms of the outcomes in the period before the centralisations. The synthetic controls will use a weighted combination of Trusts from the rest of England to approximate pre-centralisation outcomes in London Cancer and Manchester Cancer. Trends in outcomes in London Cancer, Manchester Cancer and their synthetic controls will then be compared over time using an adapted version of the regression model described above.

We will also use patient level regression analysis to relate the intermediate outcomes to the clinical outcomes. We will regress the outcomes against the care processes, the latter being included separately in individual models, and all together in a single model. In these models, we will control for gender, age, interactions between age and gender, cancer diagnosis, Charlson comorbidity index, presence of comorbidities, ethnic group, deprivation and rural-urban classification.

#### Cost-effectiveness

We will construct cost-effectiveness models to test whether centralisations reflect good value for money 30 days and 1 year post-surgery. Before and after decision analytic models will be constructed, with a different model for each type of surgical cancer centralisation per region. Where possible, we will construct a decision analytic model of an urban region in England that has not been centralised as a control, using the synthetic controls described above. The models will be used to calculate NHS and personal social service costs and outcomes of surgery pre- and post-centralisation, providing policy makers, commissioners and providers with information regarding value for money of centralising specialist cancer surgical services. We will include information and descriptive statistics on surgery, in-patient stay, follow-up, readmission, centralisation and implementation resource use and costs. We will report any costs available from providers or commissioners. If this information is not available, national published sources will be used. Special attention will be paid to analysis of fixed and variable costs and where assets have been purchased versus staff costs, to ensure an accurate mean cost per patient.

Outcomes will be modelled as quality-adjusted life-years (QALYs). We will assess the feasibility of calculating QALYs from patient-reported and clinical outcome measures and, if this is not forthcoming, utility scores for calculating QALYs will be obtained from the CEA registry [56]. Cost-effectiveness will be calculated as the mean cost difference of centralisation (after minus before), divided by mean difference in outcomes, to give incremental cost-effectiveness ratios [63]. In addition to reporting the mean incremental costs per QALY gained, we will report the mean incremental cost per change in outcome for each cost-effectiveness model performed using outcome(s) chosen based on the DCE ranking results. We will conduct deterministic and probabilistic sensitivity analyses to explore effects of uncertainty and the effects of using national versus local values from providers or commissioners [64]. Cost-effectiveness acceptability curves will be created comparing the net monetary benefit (willingness to pay for the change in outcome, minus the incremental cost), for each of the centralisation options. We will assess the feasibility of constructing before and after life-time models for each cancer centralisation, extrapolating the results of survival and readmission data described above. We will also assess the feasibility of calculating the cost to primary care of the different centralisation models. However, this is unlikely to be viable, owing both to issues associated with accessing the necessary data, and to the additional resources that would be required to collect this information.

#### Data synthesis

Studying centralisation of four pathways across two areas allows development and testing of theories on how change processes interact with the context in which they take place. A multiple case study approach—in this case, the governance of the Manchester Cancer and London Cancer centralisations, and their implementation and impact within services—allows the analysis of different organisational contexts and different degrees of centralisation. We will combine findings from different study components to test and further develop our conceptual framework (Fig. 2); in particular, we will provide evidence on ways in which clinically-led networks contribute to major system change in complex organisational settings (e.g. the extent to which networks facilitate working across organisational and professional boundaries). Our examination of the outcomes of the centralisations (what works and at what cost) will be grounded in our understanding of the planning and implementation of the changes (‘how and why’).

#### Exploring generalisability to other contexts

Towards the end of this study, we will hold a stakeholder workshop to share findings with policy makers, clinicians, managers, patients and carers from a range of clinical settings across England. Attendees will discuss how our findings might apply to other contexts and how best to take our lessons forward, e.g. identifying translational obstacles and enablers.

## Discussion

Through the DCE, this study will provide valuable insights on key stakeholders’ priorities in relation to changes of this kind. We will present an analysis of the impact of these centralisations on care provision, clinical outcomes, cost-effectiveness and patient experience. However, such findings alone are of limited benefit, as they leave unanswered the important questions of how impacts were achieved (or not) and which factors were influential. We will conduct in-depth qualitative analysis of how the new models of care were planned and implemented, and their impact, using a framework identifying key components of major system change developed in previous research on acute stroke service centralisation [31]. We will test and develop this framework in a different healthcare context and contribute to understanding of how networks operate across complex organisational settings and influence major system change. Our focus on contextual factors will support generalisability beyond the specialist cancer surgery settings studied. We will thus generate lessons for potential future centralisations of specialist services, in terms of engaging key stakeholders, planning and implementing change, and potential impact on quality and outcomes of care.

This study faces a number of challenges. For the DCE, we require at least 400 responses, covering patients, healthcare professionals and the public. To maximise responses, we will publicise the survey actively and work with Quality Health (who runs the NCPES) to obtain a balanced sample in a manner that does not threaten patient privacy.

Certain aspects of the qualitative analysis, especially in relation to London Cancer, will be retrospective. We have addressed this issue by careful stakeholder sampling and collecting contemporaneous documentation. The relative recency of the London changes means the centralisations remain a relevant issue.

For the analysis of outcomes, an issue relates to the distinction between primary and secondary outcome measures. As noted, the primary outcomes used to power the analysis were selected with clinician team members, though other measures were also identified as important. While we recognise the value of primary outcomes in demonstrating feasibility and usefulness of the analysis, we will analyse a range of outcome measures and consider the distinction between ‘primary’ and ‘secondary’ measures somewhat arbitrary. Secondly, a practical challenge will be assembling and analysing the data in a timely manner, especially for Manchester Cancer as the changes there have yet to occur. This will also affect the cost-effectiveness analysis, which depends on data and results from this quantitative analysis.

Further, the cost-effectiveness analysis might encounter difficulties in obtaining accurate information on the time people spent working on the centralisations. This is common to many implementation studies, as people find it hard to quantify exactly the time they dedicate to a particular project, given overlap with other projects or day-to-day working. However, evaluating the changes in real-time and collecting data prospectively increases the likelihood of our obtaining these data.

There are significant drives to centralise healthcare services, but the evidence available in relation to such changes is limited. This study will generate important lessons—in terms of stakeholder preferences for centralisations, how change is planned and implemented and its impact on care provision, clinical outcomes and costs—that will be of value to planners of service centralisation, both in the English NHS and internationally.
